# Transcriptome Sequencing of* Gynostemma pentaphyllum* to Identify Genes and Enzymes Involved in Triterpenoid Biosynthesis

**DOI:** 10.1155/2016/7840914

**Published:** 2016-12-14

**Authors:** Qicong Chen, Chengtong Ma, Jieying Qian, Xiuwan Lan, Naixia Chao, Jian Sun, Yaosheng Wu

**Affiliations:** Key Laboratory of Biological Molecular Medicine Research of Guangxi Higher Education, Department of Biochemistry and Molecular Biology, Guangxi Medical University, Nanning, Guangxi, China

## Abstract

*G. pentaphyllum *(*Gynostemma pentaphyllum*), a creeping herbaceous perennial with many important medicinal properties, is widely distributed in Asia. Gypenosides (triterpenoid saponins), the main effective components of* G. pentaphyllum*, are well studied. FPS (farnesyl pyrophosphate synthase), SS (squalene synthase), and SE (squalene epoxidase) are the main enzymes involved in the synthesis of triterpenoid saponins. Considering the important medicinal functions of* G. pentaphyllum*, it is necessary to investigate the transcriptomic information of* G. pentaphyllum* to facilitate future studies of transcriptional regulation. After sequencing* G. pentaphyllum*, we obtained 50,654,708 unigenes. Next, we used RPKM (reads per kilobases per million reads) to calculate expression of the unigenes and we performed comparison of our data to that contained in five common databases to annotate different aspects of the unigenes. Finally, we noticed that FPS, SS, and SE showed differential expression of enzymes in DESeq. Leaves showed the highest expression of FPS, SS, and SE relative to the other two tissues. Our research provides transcriptomic information of* G. pentaphyllum* in its natural environment and we found consistency in unigene expression, enzymes expression (FPS, SS, and SE), and the distribution of gypenosides content in* G. pentaphyllum*. Our results will enable future related studies of* G. pentaphyllum*.

## 1. Introduction

*Gynostemma pentaphyllum* (Thunb.) Makino is a kind of creeping herbaceous perennial that is distributed in Asia.* Gynostemma pentaphyllum* (*G. pentaphyllum*) grows in many places of China, including Guangxi, Guangdong, Fujian, Guizhou, Yunnan, Hubei, Anhui, Hebei, Jiangsu, Henan, Shandong, Sichuan, and Shanxi and in Taiwan.* G. pentaphyllum* also grows in neighboring countries such as Bangladesh, India, Indonesia, Japan, Republic of Korea, and Malaysia (data was obtained from the Checklist of South China Botanical Garden) [1]. Gypenosides (triterpenoid saponins), the major effective components of* G. pentaphyllum*, have various bioactivities that explain the extensive application of* G. pentaphyllum* in natural medicines [2–13]. For instance, the gypenosides exhibit a hypoglycemic effect by increasing the secretion of insulin [11–13]. Other functions like anticancer function, anti-inflammatory function, antianxiety function, blood fat-reducing, liver cells-protecting, neuroprotection, and immunoprotection have also been reported [2–10].

**Figure 1:**
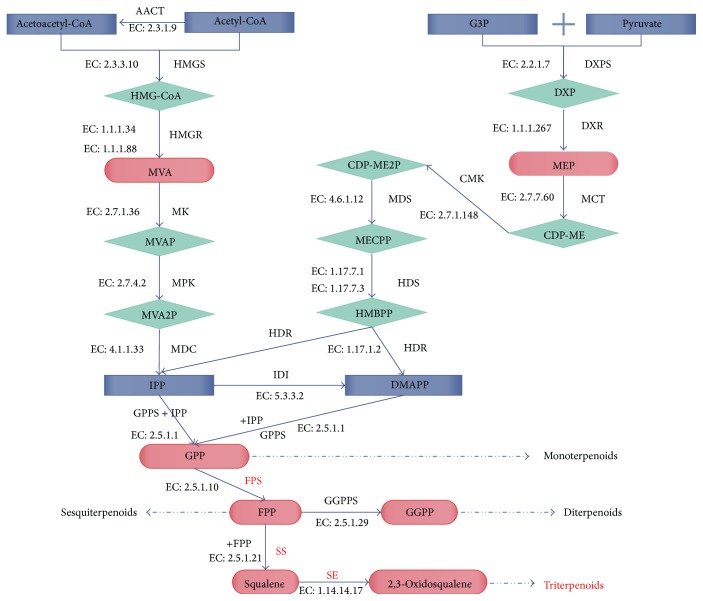
Triterpenoid synthesis pathway. Note: AACT: acetyl-CoA C-acetyltransferase; HMGS: hydroxymethylglutaryl-CoA synthase; HMG-CoA: hydroxymethylglutaryl-CoA; HMGR: hydroxymethylglutaryl-CoA reductase; MVA: mevalonate; MK: mevalonate kinase; MVAP: mevalonate phosphate; MPK: mevalonate phosphate kinase; MVAPP: mevalonate diphosphate; MDC: mevalonate diphosphate decarboxylase; G3P: D-glyceraldehyde-3-phosphate acetaldehydetransferase; DXPS: 1-deoxy-D-xylulose-5-phosphate synthase; DXP: 1-deoxy-D-xylulose-5-phosphate; DXR: 1-deoxy-D-xylulose-5-phosphate reductoisomerase; MEP: 2-C-methyl-D-erythritol 4-phosphate; MCT: 2-C-methyl-D-erythritol 4-phosphate cytidylyltransferase; CDP-ME: 4-(cytidine 5′-diphospho)-2-C-methyl-D-erythritol; CDP-ME2P: 2-phospho-4-(cytidine 5′-diphospho)-2-C-methyl-D-erythritol; MECPP: 2-C-methyl-D-erythritol 2,4-cyclodiphosphate; HDS: l-hydroxy-2-methyl-butenyl-4-diphosphate synthase; HMBPP: l-hydroxy-2-methyl-2-butenyl-4-diphosphate; HDR: 4-hydroxy-3-methylbut-2-enyl diphosphate reductase; IPP: isopentenyl-PP; IDI: isopentenyl-diphosphate delta-isomerase; DMAPP: dimethylallyl-PP; GPS: geranyl-diphosphate synthase; GPS: geranyl-diphosphate synthase; GPPS: geranylgeranyl diphosphate synthase; GPP: geranylgeranyl diphosphate; FPS: farnesyl diphosphate synthase; GGPPS: geranylgeranyl diphosphate synthase; GGPP: geranylgeranyl diphosphate; SS: squalene synthase; and SE: squalene epoxidase.

Gypenosides are secondary metabolites in the synthesis pathway of triterpenoids. Mevalonate or isoprenoid are the precursors in the beginning of the pathway of triterpenoid synthesis, which we refer to as the MVA (mevalonic acid) or MEP (methylerythritol phosphate) pathway (Figure 1) [14–16]. The synthesis pathway of the triterpenoids can be decomposed into three parts: (1) the synthesis of IPP (isopentenyl pyrophosphate) or DMAPP (dimethylallyl pyrophosphate); (2) the synthesis and cyclization of the squalene; and (3) the functionalization reaction that proceeds with complexity of the squalene (Figure 1). FPS (farnesyl pyrophosphate synthase), SS (squalene synthase), and SE (squalene epoxidase) were previously identified as the main enzymes involved in the synthesis of triterpenoid saponins [17–20]. FPS, SS, and SE are required for the synthesis and cyclization of the squalene that combines two sesquiterpenoids into one triterpenoid (C15 + C15 = C30) [16, 21]. After this step, triterpenoids can be transformed into many isoform types like* protosteryl type* (chair-chair-chair-boat conformations)*, dammarenyl type *(chair-chair-chair-boat conformations)*, cadinyl type* (chair-chair-chair-boat conformations), and* hopene and tetrahymanol *(chair-chair-chair-chair conformations or chair-chair-chair-boat conformations) [21].

Like the ginsenosides, gypenosides (triterpenoids) in* G. pentaphyllum* have various and vital applications in medicine and health [22]. However, gypenosides showed much higher heterogeneity when compared with ginsenosides and more than 169 kinds of gypenosides were found in* G. pentaphyllum* [23–28]. In other words, more than five times the number of triterpenoid saponins was found in* G. pentaphyllum* relative to* Panax ginseng (P. ginseng)*. It was interesting that* G. pentaphyllum* has such diversity in triterpenoids compared to other plants [26]. This is likely related to different expression of genes and enzymes involved in the synthesis pathway of triterpenoids. Nowadays, transcriptomic sequencing (RNA sequencing) is a more and more popular tool to explore transcriptomic process [29–37], because RNA sequencing has several advantages relative to DNA sequencing like lower fee, higher efficiency, more advanced features, and so forth [36–39]. In 2011, Sathiyamoorthy Subramaniyam analyzed the transcriptome of* G. pentaphyllum* related to the synthesis pathway of triterpenoids. However, this article had two important limitations. First, the samples used for sequencing only included two tissues (leaves and roots) and* G. pentaphyllum* that was sampled and sequenced was planted in water and not in its natural environment [32]. This is important because* G. pentaphyllum* exhibits great phenotypic diversity in different environments because of its strong adaptability [33, 40–44]. Additionally, although the author showed sequencing data in the paper, no association analysis among unigenes expression, enzyme expression, or the distribution of gypenosides content of* G. pentaphyllum* was determined. In 2015, Zhao et al. identified EST-SSR makers by analyzing the sequencing data of two species of* Gynostemma* (Cucurbitaceae) [33]. In that article, the tissues were natural and complete, but the three tissues (young leaves, flowers, and immature seeds) from each kind of* G. pentaphyllum* were mixed up together to extract RNA for constructing cDNA to sequence. In other words, the sequencing data and related information in that article were a mixed result and these results could not be classified by tissues. Therefore, to address this deficiency of knowledge, we collected* G. pentaphyllum* in natural environment and sequenced its transcriptome separately by Illumina's NextSeq 500.

## 2. Materials and Methods

### 2.1. Sample Collection and Preparation

*G. pentaphyllum* used for sequencing was planted in the Medicinal Plant Garden of Guangxi Traditional Chinese Medical University, Nanning City, Guangxi Autonomous Region, China. In July 2015, we harvested* G. pentaphyllum* after identification by Mr. Yilin Zhu (Guangxi Traditional Chinese Medical University). Fibrous roots, leaves, and stems were separately collected and cleaned and removed of impurities like soil (biological repeat of collections of each tissue was three times) (Figure S1–S5 in Supplementary Material available online at http://dx.doi.org/10.1155/2016/7840914). Finally, the samples were saved in cryotubes and submerged in liquid nitrogen immediately.

### 2.2. Illumina Sequencing

The plant tissue sample of* G. pentaphyllum* was sent to* Personalbio Company *(Shanghai City, China) for transcriptome sequencing using Next-Generation Sequencing (NGS) technology based on the sequencing platform of Illumina's NextSeq 500. First, the mRNA was cleaved into little segments after treatment with chemical reagents and high temperature. Next, the segments were used to construct a cDNA library that was sequenced by paired end (PE) reads.

### 2.3. Unigene Assembly

Trinity (r20140717, k-mer 25 bp) professional software was used to assemble the RNA sequence [45]. First, high-quality sequences were constructed into a short-sequence library with length of k-mer. Next, primary contig sequences were obtained by the extension of the short-sequence library using overlaps with a k-mer-1 length. Next, primary contig sequences were categorized by their overlaps and categorical contigs were constructed into the De Bruijn graph. Based on the recognition rate of reads in each category, transcript sequences were restored by the contigs. After assemblage by Trinity, BLAST (version 2.2.30+) was used to compare the assembled sequences with reference sequences in NCBI (National Center for Biotechnology Information) nonredundant protein (NR) sequences to determine the best comparison results. Finally, sequences with the same gi number were classified as the same unigene and the longest sequence was regarded as the representative sequence of that unigene [46].

### 2.4. Analysis of Unigene Expression

RPKM (reads per kilobases per million reads) was used to calculated unigene expression of* G. pentaphyllum* and the calculation method of RPKM is described below [47]. Before we calculated the unigene expression, we need to process the read count of unigenes with Bowtie 2 (2.2.4, default setting) [48]. The RPKM density distribution generally reflected the pattern of gene expression. Typically, unigenes with medium expression cover the majority of the area under the curve (AUC) in the density distribution of RPKM (as drawn with the density function of software R). Oppositely, unigenes with higher or lower expression occupy the minority of AUC. DESeq (version 1.18.0) software was used to analyze the differential expression of unigenes in our study [49]. The expression of unigenes was compared by the fold change (fold change > 2) and its significance (*p* value < 0.05). The final result was displayed by Venn diagram.

(1) RPKM=total  exon  readsmapped  readsmillions∗exon  lengthKB

### 2.5. Functional Annotation

After categorization, unigenes were annotated for functions using five databases: NCBI nonredundant protein (NR) sequences, Gene Ontology (GO) [50, 51], Kyoto Encyclopedia of Genes and Genome (KEGG) [52, 53], evolutionary genealogy of genes: Nonsupervised Orthologous Groups (eggNOG), [54] and Swiss-Prot [55, 56].

### 2.6. Analysis of the Distribution of Gypenosides Content in* G. pentaphyllum*

First, dried powder of the sample (about 15 mg) was mixed with 10 mL of extraction solvent (ethanol containing 5.0% pure water) and was processed by a continued supersonic treatment for 30 minutes. Second, the mixed solvent was evaporated to dryness using a rotary evaporator. The dry gypenosides were redissolved in 5.0 mL hot water (50°C). Third, this solution was applied to a chromatography column containing D101 macroporous resin and allowed to stand for 20 minutes. Fourth, pure water was used to rinse unbound material from the macroporous resin, while extraction solvent (ethanol containing 50% pure water) was used to remove the gypenosides form column. Fifth, the solvent of gypenosides was brought up to 5.0 mL and processed with a color reaction by vanillin. We then detected the absorbance of the solvent (after color reaction) by UV-Vis Spectrophotometer at a wavelength of 584 nm. Finally, we used Panaxadiol (C_30_H_52_O_3_) as a standard sample to calculate the actual content of gypenosides in samples by the standard curve method.

## 3. Result

### 3.1. Overview of the Sequencing and Assembly

The mRNA of* G. pentaphyllum* was cut into many small segments to construct a cDNA library. After sequencing the cDNA library, we obtained 352,999,296 original reads. However, this set of original reads also contained a lot of adapters and low-quality sequences, so 103,500,643 reads were filtered out leaving 249,488,643 clean reads of high quality. Next, 1,119,964 contigs with a total length of 249,488,543 bp were assembled by the overlaps of the original reads and we used these contigs to restore the transcriptome sequences. In the next step, we harvested 159,858,904 transcriptome sequences and used BLAST (Basic Local Alignment Search Tool) for all transcriptome sequences in the NR database. The transcriptome sequence with the highest score in BLAST was saved and the transcriptome sequences with same gi number were categorized as coming from the same unigene. Finally, we obtained 50,654,708 unigenes with a mean length of 755 bp. The overview of sequences is presented in Table 1.

### 3.2. Result of Annotation

We used five databases, NR, GO, KEGG, eggNOG, and Swiss-Port, to annotate unigenes for functions (Figure S6–S9). The overview of annotation is listed in Table 2. The result of each annotation is provided in the support file. Generally speaking, eggNOG showed an identification rate of 96.57% and KEGG displayed the lowest identification rate of 8.77% when comparing unigenes with the reference sequences. Based on GO annotation, the unigenes were categorized into different categories based on different functions and GO Slim displayed general characteristic about the distribution of the unigenes. Then, we used the eggNOG database to explore the biological function of protein in more detail because eggNOG classifies different protein sequences into a more detailed directory. Similarly, Swiss-Port was also used for annotation of the protein sequences, and Swiss-Port builds on eggNOG and provided more detailed structural information about the protein. Finally, KEGG is the last but most important database we used to annotate enzymes, since the KEGG pathway annotation showed us the network of the intermolecular reaction. This allows determination of the enzymes that are located in the synthesis pathway of triterpenoid saponins.

### 3.3. Expression of Unigenes

**Figure 2:**
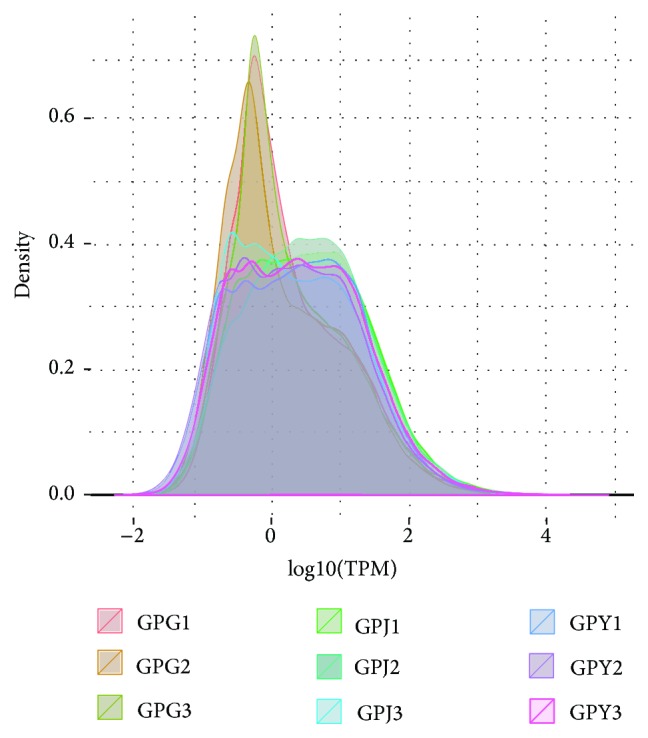
Density distribution of RPKM. Note: GPG: fibrous roots; GPJ: stems; GPY: leaves; RPKM: reads per kilobases per million reads.

**Figure 3:**
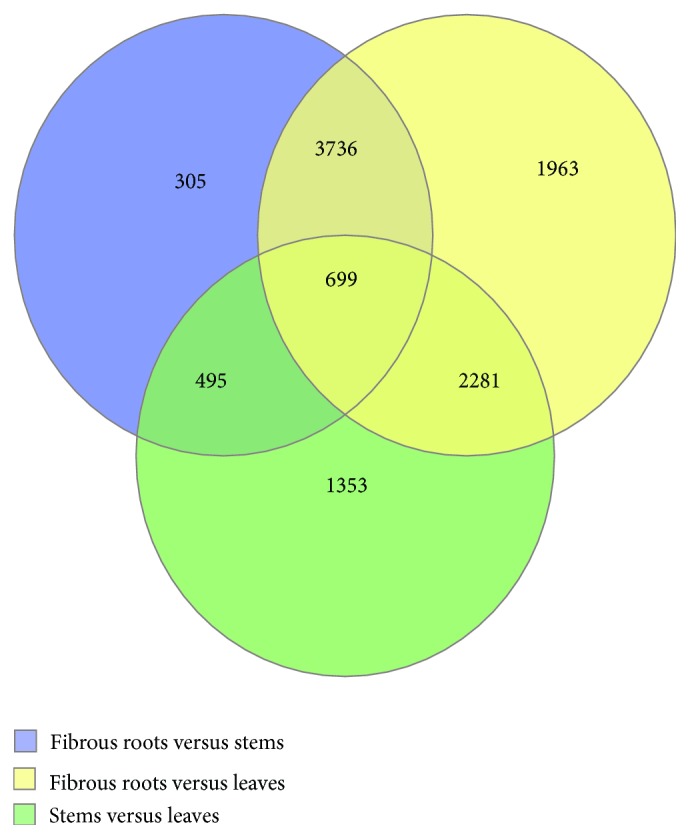
Venn diagram of differential expression of unigenes.

RPKM is a normalization method to calculate gene expression and we used the density distribution of the RPKM to show the expression of unigenes. The map of the density distribution showed that our unigenes expression conformed to standards because unigenes with mid-range expression occupied the majority of AUC (area under the curve) and unigenes with lower or higher expression were the minority of AUC (Figure 2). In the density distribution, unigenes in fibrous roots showed much higher expression than in the stems and leaves. Although stems and leaves showed similar unigene expression, stems had slightly higher unigene expression than the leaves. We analyzed the result of expression of the unigenes of FPS, SS, SE, and *β*-AS (beta-amyrin synthase) in Table 3. Noticeably, the unigenes that encoded FPS, SS, SE, and *β*-AS showed the highest expression in leaves and the lowest expression in fibrous roots. In the unigene expression of *β*-AS, the leaves showed almost 125 times higher expression than in the fibrous roots. The unigenes of FPS, SS, SE, and *β*-AS showed higher expression in the stems than in the fibrous roots. Based on this sequencing data, we detected the differential expression of unigenes using the software DESeq. We obtained the upregulated and downregulated unigenes in the pairwise comparison among the data from the fibrous roots, stems, and leaves (Table 4). We also determined the unigenes that showed differential expression in all samples. We used Venn diagram function in software R to describe the general distribution of unigenes with differential expression (Figure 3). Combining the data in Table 4 and Figure 3, we concluded that 10832 unigenes showed differential expression. Additionally, 699 unigenes displayed differential expression in all samples, while 6512 unigenes showed differential expression in the pairwise comparison of samples.

### 3.4. Content Distribution of Gypenosides in* G. pentaphyllum*

A UV-Vis Spectrophotometer was used to detect the content distribution of gypenosides in* G. pentaphyllum. *Leaves had the highest content (3.189%) of gypenosides of all samples (Table 5), and the content of gypenosides in stems (0.365%) or fibrous roots (0.172%) was much lower than the leaves. A correlation coefficient (*R* ^2^) of 0.996 in the standard curve indicates that our result was accurate and reliable (Figure S10).

### 3.5. Expression of Enzymes in the Synthesis Pathway of Triterpenoids

**Figure 4:**
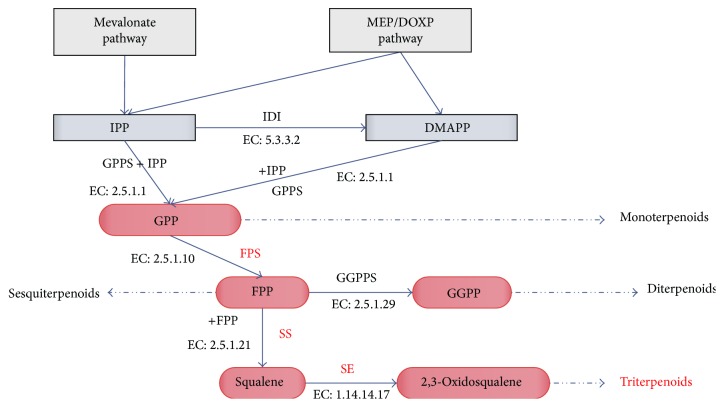
FPS, SS, and SE in the synthesis pathway of triterpenoids. Note: IPP: isopentenyl-PP; IDI: isopentenyl-diphosphate delta-isomerase; DMAPP: dimethylallyl-PP; GPS: geranyl-diphosphate synthase; GPPS: geranylgeranyl diphosphate synthase; GPP: geranylgeranyl diphosphate; FPS: farnesyl diphosphate synthase; GGPPS: geranylgeranyl diphosphate synthase; GGPP: geranylgeranyl diphosphate; SS: squalene synthase; and SE: squalene epoxidase.

**Figure 5:**
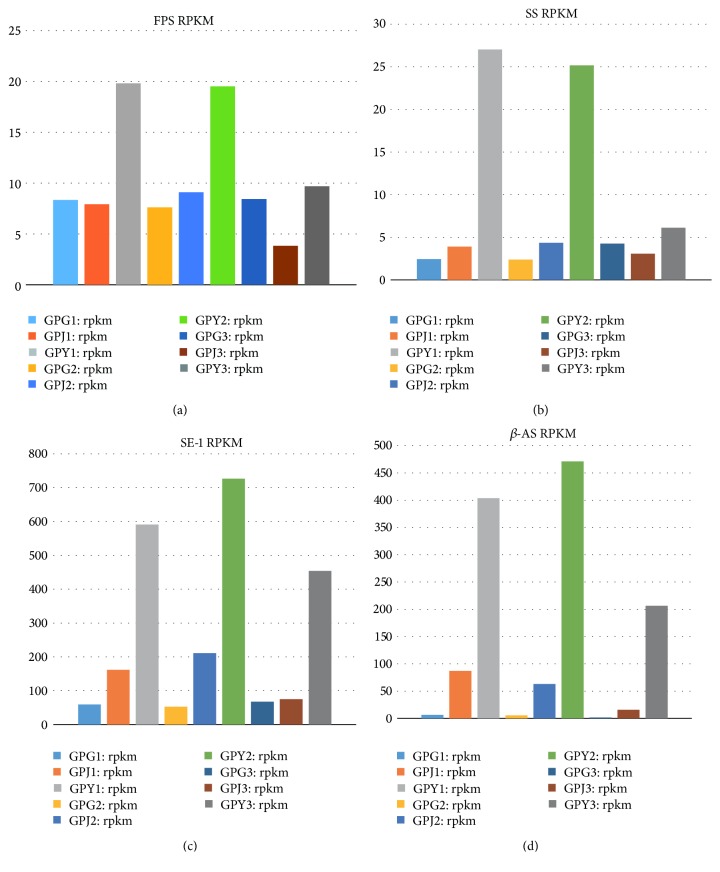
RPKM of FPS, SS, SE, and *β*-AS. Note: (a) RPKM of FPS; (b) RPKM of SS; (c) RPKM of SE-1; (d) RPKM of *β*-AS; GPG: fibrous roots; GPJ: stems; GPY: leaves; RPKM: reads per kilobases per million reads.

Based on the KEGG Pathway, a more detailed result about the enzymes expression in the triterpenoids synthesis was obtained. We categorized related enzymes into three parts according to the synthesis pathway of the triterpenoids: (1) enzymes involved in the synthesis of IPP or DMAPP, (2) enzymes involved in the synthesis and cyclization of squalene, and (3) enzymes in the squalene functionalization reaction (Figure 1). We focused on FPS, SS, and SE because these three enzymes play an important role in the synthesis and cyclization of triterpenoids (Figure 4) [17–19]. The results are shown in Table 6 and Figure 5. We found three noticeable results. First, in the comparison of FPS, only one comparison between the fibrous roots and leaves was found. Leaves showed higher expression of FPS compared to the fibrous roots. Second, in the comparison of SS, two comparisons were found and the result showed that leaves had higher expression than the fibrous roots and stems. Third, in the comparison of SE, leaves showed higher expression than the stems and fibrous roots. The fibrous roots showed higher expression of SE than the stems.

## 4. Discussion

*G. pentaphyllum* is a creeping herbaceous perennial with medicinal properties used in traditional Chinese medicine. Triterpenoid saponins, the main effective components of* G. pentaphyllum*, have been widely studied [2–13, 23, 24]. In this study, we obtained transcriptome information using RNA sequencing, a technique that exhibits higher efficiency and is less expensive than DNA sequencing [38]. We analyzed the associations of unigene expression, enzyme (the output of the unigenes) expression, and the content distribution of gypenosides (enzyme output) after functional annotation (Table 2), RPKM calculating (Table 3), and measurement of gypenosides content (Table 5).

We found that the expressions of unigenes and enzymes were positively associated with the distribution of gypenosides content. Generally speaking, unigenes and enzyme expression (FPS, SS, and SE) in the samples (fibrous roots, stems, and leaves) determined to the distribution of gypenosides content. Higher expression of unigenes and enzymes (encoded by unigenes) caused the higher content of enzymes' production (gypenosides), and the lower expression of unigenes and enzymes caused less enzyme production. This consistent result could facilitate future studies of other secondary metabolites in* G. pentaphyllum*. Since* G. pentaphyllum* has wide applications for health and medicine, it is essential to identify the secondary metabolites with various and vital medicinal functions.

One interesting finding was the observed differences between general expression and individual expression of unigenes (Figure 2 and Table 3). A group of specific unigenes (FPS, SS, and SE) located in a special pathway like triterpenoids synthesis could increase their expression to a much higher level as required for a special physiological activity like triterpenoid synthesis. This obvious difference in expression may result from changes in the regulation of transcription. It is currently a hot topic to study the differential expression of the transcriptome and the regulation of transcription of plants in response to stresses of a special environment or other genetic factors [57–63]. Another interesting observation was that the transcriptome sequences of* G. pentaphyllum* determined in our study showed high similarity to the transcriptome sequences related to bitterness in cucumber as reported previously [64]. We downloaded the available mRNA sequences of related enzymes from that article and blasted them against the unigene sequences of* G. pentaphyllum*. The BLAST result was surprising, as all thirteen available sequences related to bitterness in that article showed a high degree of similarity to the specific unigene sequences of* G. pentaphyllum*. This high similarity predicted that genes related to the biosynthesis, regulation, and domestication of bitterness in cucumber may also be present in* G. pentaphyllum*.* G. pentaphyllum* also has two tastes (sweet and bitter) and this difference of taste may be caused as in cucumber. The taste of* G. pentaphyllum* from bitter to sweet predicts that* G. pentaphyllum* may change in response to domestication and contain a similar mutation. A further genetic exploration of the domestication of* G. pentaphyllum* may provide understanding of its changed taste.

Although this study was not the first report of the transcriptome information for* G. pentaphyllum* using sequencing, we corrected the limitations of the previous study and enriched the analysis of triterpenoid synthesis of* G. pentaphyllum*.* G. pentaphyllum* used for analysis in our study was planted in a natural environment and three common kinds of plant tissues (fibrous roots, stems, and leaves) were used to provide tissue samples for sequencing. Together with the sequencing, an exploration of the distribution of gypenosides content was performed to confirm the analysis of enzymes and unigenes. We found a positive association of unigene expression, enzyme expression, and the distribution of gypenosides. Our study will facilitate more genetic studies examining the regulation of transcription and the change of bitterness in* G. pentaphyllum*.

## 5. Conclusions

To provide more complete and high-quality transcriptional information of natural* G. pentaphyllum*, we used RNA sequencing technology to sequence the transcriptome of* G. pentaphyllum.* We found a positive association of unigene expression, enzyme expression, and the distribution of gypenosides. Our results will enable future related studies of* G. pentaphyllum*.

## Supplementary Material

Figure S1. *G. pentaphyllum* (Sample) 01. Description: This is the picture of intact *G. pentaphyllum* in our study.
Figure S2. G. pentaphyllum (Sample) 02. Description: This is the picture of intact *G. pentaphyllum* in our study.
Figure S3. Leaves (Sample). Description: This is the picture of leaves sample of *G. pentaphyllum* in our study.
Figure S4. Stems (Sample). Description: This is the picture of stems sample of *G. pentaphyllum* in our study.
Figure S5. Fibrous Roots (Sample). Description: This is the picture of fibrous roots sample of *G. pentaphyllum* in our study.
Figure S6. The general result of annotation. Abbreviations: NR: Nonredundant protein sequences; GO: Gene Ontology; KEGG: Kyoto Encyclopedia of Genes and Genome; eggNOG: Evolutionary genealogy of genes: Nonsupervised Orthologous Groups.
Figure S7. The result of GO Slim. Abbreviation: GO Slim: Cut-down versions of the GO ontologies.
Figure S8. The result of eggNOG annotation.
Figure S9. The result of KEGG annotation.
Figure S10. The standard curve of absorbance.

## Figures and Tables

**Table 1:** Overview of the sequencing and assembly.

Categories	Description	Number
Total reads	Total number of reads (RAW)	356,311,342
	Number of clean reads	352,999,296
Contigs	Total length (bp)	249,488,543
	Sequence number	1,119,964
	Max. length (bp)	13,870
	Mean length (bp)	223
	GC%	48
Transcriptome	Total length (bp)	159,858,904
	Sequence number	319,480
	Max. length (bp)	11,670
	Mean length (bp)	500
	GC%	46
Unigenes	Total length (bp)	50,654,708
	Sequence number	67,068
	Max. length (bp)	11,670
	Mean length (bp)	755
	GC%	44

**Table 2:** Overview of annotation.

Annotation in database	Unigene number	Percentage (%)
NR	67,068	100
GO	40,623	61
KO	5,884	9
eggNOG	64,768	97
Swiss-Prot	55,429	83
In all databases	5,031	7.5

**Table 3:** RPKM of unigenes (FPS, SS, SE, and *β*-AS) in samples.

Enzyme	Unigene ID	RPKM of sample 1			RPKM of sample 2			RPKM of sample 3		
		Fibrous roots	Stems	Leaves	Fibrous roots	Stems	Leaves	Fibrous roots	Stems	Leaves
FPS	c118250_g1_i1	8.35	7.93	19.81	7.61	9.11	19.53	8.44	3.84	9.7
SS	c136108_g1_i1	2.44	3.91	27.03	2.39	4.36	25.18	4.26	3.06	6.12
SE-1	c127030_g2_i1	59.23	161.95	591.15	53.05	211.1	726.75	67.54	74.84	454.22
SE-2	c113536_g2_i1	0.79	0.95	5.66	0.26	0.94	2.87	0.6	0.52	2.7
*β*-AS	c70785_g1_i1	6.49	86.98	403.79	5.5	62.95	471.36	1.66	15.79	206.31

Note: FPS: farnesyl pyrophosphate synthase; SS: squalene synthase: SE: squalene epoxidase; *β*-AS: beta-amyrin synthase.

**Table 4:** Overview of the upregulated and downregulated unigenes.

Case	Control	Upregulated unigenes		Downregulated unigenes		Total DE unigenes	
		Number	%	Number	%	Number	%
GPG	GPJ	3476	5.18	1759	2.62	5235	7.81
GPG	GPY	5590	8.33	3089	4.61	8679	12.94
GPJ	GPY	2938	4.38	1890	2.82	4828	7.2

Note: GPG: fibrous roots of *G. pentaphyllum*; GPJ: stems of *G. pentaphyllum*; GPY: leaves of *G. pentaphyllum*; DE: differential expression.

**Table 5:** Content distribution of gypenosides in *G. pentaphyllum*.

Sample	Content of gypenosides			
	Number 1	NUmber 2	Number 3	Average
Fibrous roots	0.1725%	0.1731%	0.1731%	0.1729%
Stems	0.3645%	0.3657%	0.3682%	0.3662%
Leaves	3.1887%	3.1912%	3.1931%	3.1910%

**Table 6:** Enzyme expression of FPS, SS, SE, and *β*-AS.

Full name	Unigene ID	Short name	EC number	G versus J	G versus Y	J versus Y
Farnesyl diphosphate synthase	c118250_g1_i1	FPS	EC: 2.5.1.1/2.5.1.10		GPY up	
Squalene synthase	c136108_g1_i1	SS	EC: 2.5.1.21		GPY up	GPY up
Squalene epoxidase	c127030_g2_i1	SE	EC: 1.14.13.132	GPJ up	GPY up	GPY up
					GPY up	GPY up
*β*-amyrin synthase	c70785_g1_i1	*β*-AS	EC: 5.4.99.39	GPJ up	GPY up	GPY up

Note: GPG or G: fibrous roots of *G. pentaphyllum*; GPJ or J: stems of *G. pentaphyllum*; GPY or Y: leaves of *G. pentaphyllum*.

## Abbreviations

*G. pentaphyllum*: * Gynostemma pentaphyllum* (Thunb.) Makino
FPS: Farnesyl pyrophosphate synthase
SS: Squalene synthase
SE: Squalene epoxidase
RPKM: Reads per kilobases per million reads
NR: Nonredundant protein sequences
GO: Gene Ontology
eggNOG: Evolutionary genealogy of genes: Nonsupervised Orthologous Groups
KEGG: Kyoto Encyclopedia of Genes and Genome
MVA: Mevalonic acid
MEP: Methylerythritol phosphate
IPP: Isopentenyl pyrophosphate
DMAPP: Dimethylallyl pyrophosphate
*P. ginseng*: * Panax ginseng*
BLAST: Basic Local Alignment Search Tool
GO Slim: Cut-down versions of the GO ontologies
AUC: Area under the curve
*β*-AS: Beta-amyrin synthase
UV-Vis: Ultraviolet-visible
NGS: Next-Generation Sequencing
PE: Paired end
NCBI: National Center for Biotechnology Information
CDS: Coding sequence
RefSeq: Reference sequences
PDB: Protein database
EMBL: European Molecular Biology Laboratory
DDBJ: DNA Data Bank of Japan
KO: KEGG Ortholog.

## Data Access

The data and materials were listed in the supplementary information.
