# Supplementary material for: Transcriptome Sequencing of Gynostemma pentaphyllum to Identify Genes and Enzymes Involved in Triterpenoid Biosynthesis
Source: Int J Genomics. 2016 Dec 14;2016:7840914. doi: 10.1155/2016/7840914 (PMC5206855; doi:10.1155/2016/7840914)
Supplement: Supplementary file 1 — Figure S1. G. pentaphyllum (Sample) 01. Description: This is the picture of intact G. pentaphyllum in our study. Figure S2. G. pentaphyllum (Sample) 02. Description: This is the picture of intact G. pentaphyllum in our study. Figure S3. Leaves (Sample). Description: This is the picture of leaves sample of G. pentaphyllum in our study. Figure S4. Stems (Sample). Description: This is the picture of stems sample of G. pentaphyllum in our study. Figure S5. Fibrous Roots (Sample). Description: This is the picture of fibrous roots sample of G. pentaphyllum in our study. Figure S6. The general result of annotation. Abbreviations: NR: Nonredundant protein sequences; GO: Gene Ontology; KEGG: Kyoto Encyclopedia of Genes and Genome; eggNOG: Evolutionary genealogy of genes: Nonsupervised Orthologous Groups. Figure S7. The result of GO Slim. Abbreviation: GO Slim: Cut-down versions of the GO ontologies. Figure S8. The result of eggNOG annotation. Figure S9. The result of KEGG annotation. Figure S10. The standard curve of absorbance. [file 7840914.f1.zip › Supplementary information/Document 1. Supplementary information.docx]

Supplementary information

**Transcriptome Sequencing of** ***Gynostemma Pentaphyllum* to Identify Genes and Enzymes Involved in Triterpenoid Biosynthesis**

1. **The material used in the article**
2. **The raw files of transcriptome sequencing**
3. **The descriptions and abbreviations of the Figure S1 - S10**
4. **The material used in the article**

The bioproject of this article was signed in

<http://www.ncbi.nlm.nih.gov/bioproject/PRJNA327697>

The biosample of this article was signed in

<http://www.ncbi.nlm.nih.gov/biosample/SAMN05356211>

1. **The raw files of transcriptome sequencing**

The raw files of transcriptome sequencing were stored in the NCBI SRA database (<http://www.ncbi.nlm.nih.gov/sra/> ) and the detail information of files is listed below.

The accession of the raw files in this article was **PRJNA327697.**

| Experiment Alias | Accession |
| --- | --- |
| [JGL-G-LR1416](http://trace.ncbi.nlm.nih.gov/Traces/sra_sub/?subid=713112&action=show:EXPERIMENT&acc=SRX1941544&noheader=1#0) | SRX1941544 |
| [JGL-G-LR1424](http://trace.ncbi.nlm.nih.gov/Traces/sra_sub/?subid=713112&action=show:EXPERIMENT&acc=SRX1941545&noheader=1#0) | SRX1941545 |
| [JGL-G-LR1434](http://trace.ncbi.nlm.nih.gov/Traces/sra_sub/?subid=713112&action=show:EXPERIMENT&acc=SRX1941546&noheader=1#0) | SRX1941546 |
| [JGL-J-LR1413](http://trace.ncbi.nlm.nih.gov/Traces/sra_sub/?subid=713112&action=show:EXPERIMENT&acc=SRX1941547&noheader=1#0) | SRX1941547 |
| [JGL-J-LR1421](http://trace.ncbi.nlm.nih.gov/Traces/sra_sub/?subid=713112&action=show:EXPERIMENT&acc=SRX1941548&noheader=1#0) | SRX1941548 |
| [JGL-J-LR1431](http://trace.ncbi.nlm.nih.gov/Traces/sra_sub/?subid=713112&action=show:EXPERIMENT&acc=SRX1941549&noheader=1#0) | SRX1941549 |
| [JGL-Y-LR1409](http://trace.ncbi.nlm.nih.gov/Traces/sra_sub/?subid=713112&action=show:EXPERIMENT&acc=SRX1941550&noheader=1#0) | SRX1941550 |
| [JGL-Y-LR1418](http://trace.ncbi.nlm.nih.gov/Traces/sra_sub/?subid=713112&action=show:EXPERIMENT&acc=SRX1941551&noheader=1#0) | SRX1941551 |
| [JGL-Y-LR1428](http://trace.ncbi.nlm.nih.gov/Traces/sra_sub/?subid=713112&action=show:EXPERIMENT&acc=SRX1941552&noheader=1#0) | SRX1941552 |

1. **The descriptions or abbreviations in the Figure S1 - S10**

Figure S1. *G. pentaphyllum* (Sample) 01

Description: This is the picture of intact *G. pentaphyllum* in our study.

Figure S1. G. pentaphyllum (Sample) 02

Description: This is the picture of intact *G. pentaphyllum* in our study.

Figure S3. Leaves (Sample)

Description: This is the picture of leaves sample of *G. pentaphyllum* in our study.

Figure S4. Stems (Sample)

Description: This is the picture of stems sample of *G. pentaphyllum* in our study.

Figure S5. Fibrous Roots (Sample)

Description: This is the picture of fibrous roots sample of *G. pentaphyllum* in our study.

Figure S6. The general result of annotation

Abbreviations: NR: Nonredundant protein sequences; GO: Gene Ontology; KEGG: Kyoto Encyclopedia of Genes and Genome; eggNOG: Evolutionary genealogy of genes: Nonsupervised Orthologous Groups.

Figure S7. The result of GO Slim

Abbreviation: GO Slim: Cut-down versions of the GO ontologies.

Figure S8. The result of eggNOG annotation

None

Figure S9. The result of KEGG annotation

None

Figure S10. The standard curve of absorbance

None
