# Supplementary figures and images for: Transcriptome Sequencing of Gynostemma pentaphyllum to Identify Genes and Enzymes Involved in Triterpenoid Biosynthesis
Source: Int J Genomics. 2016 Dec 14;2016:7840914. doi: 10.1155/2016/7840914 (PMC5206855; doi:10.1155/2016/7840914)

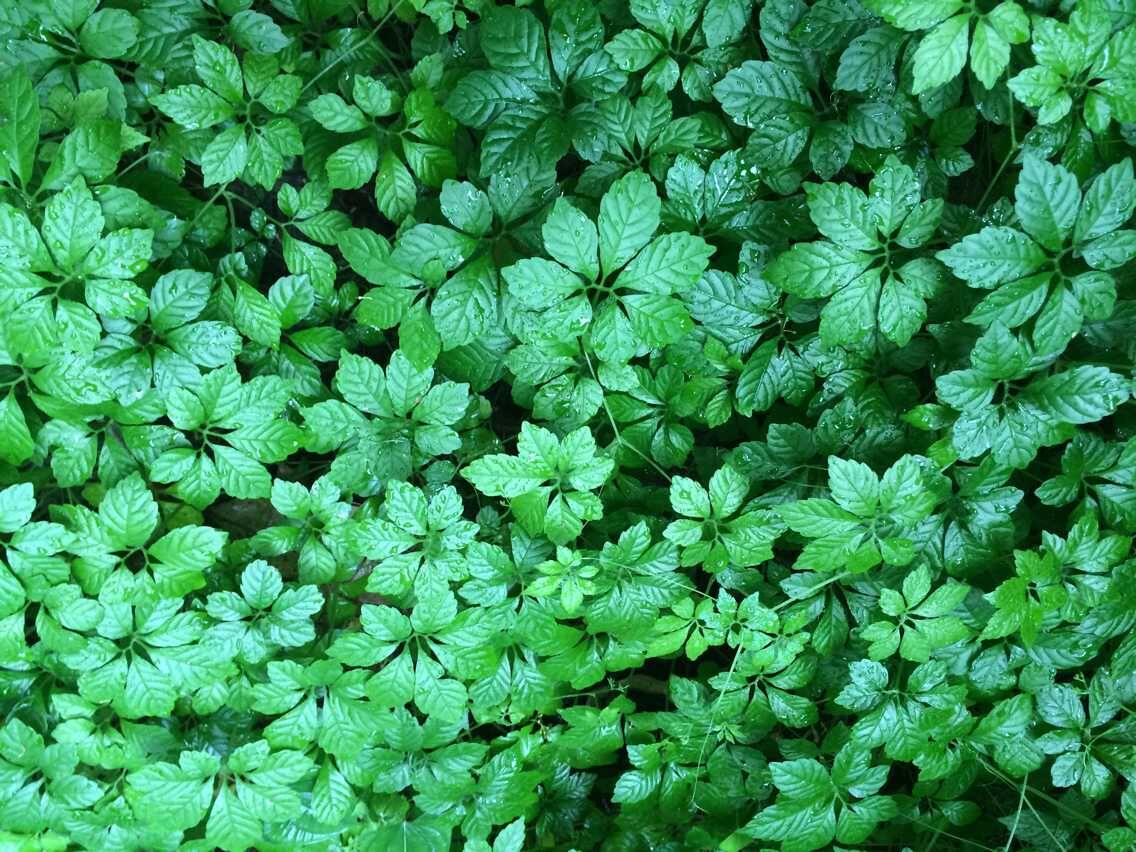

Supplement: Supplementary file 1 — Figure S1. G. pentaphyllum (Sample) 01. Description: This is the picture of intact G. pentaphyllum in our study. Figure S2. G. pentaphyllum (Sample) 02. Description: This is the picture of intact G. pentaphyllum in our study. Figure S3. Leaves (Sample). Description: This is the picture of leaves sample of G. pentaphyllum in our study. Figure S4. Stems (Sample). Description: This is the picture of stems sample of G. pentaphyllum in our study. Figure S5. Fibrous Roots (Sample). Description: This is the picture of fibrous roots sample of G. pentaphyllum in our study. Figure S6. The general result of annotation. Abbreviations: NR: Nonredundant protein sequences; GO: Gene Ontology; KEGG: Kyoto Encyclopedia of Genes and Genome; eggNOG: Evolutionary genealogy of genes: Nonsupervised Orthologous Groups. Figure S7. The result of GO Slim. Abbreviation: GO Slim: Cut-down versions of the GO ontologies. Figure S8. The result of eggNOG annotation. Figure S9. The result of KEGG annotation. Figure S10. The standard curve of absorbance. [file 7840914.f1.zip › Supplementary information/Figure S1. G. pentaphyllum (Sample) 01.jpg]

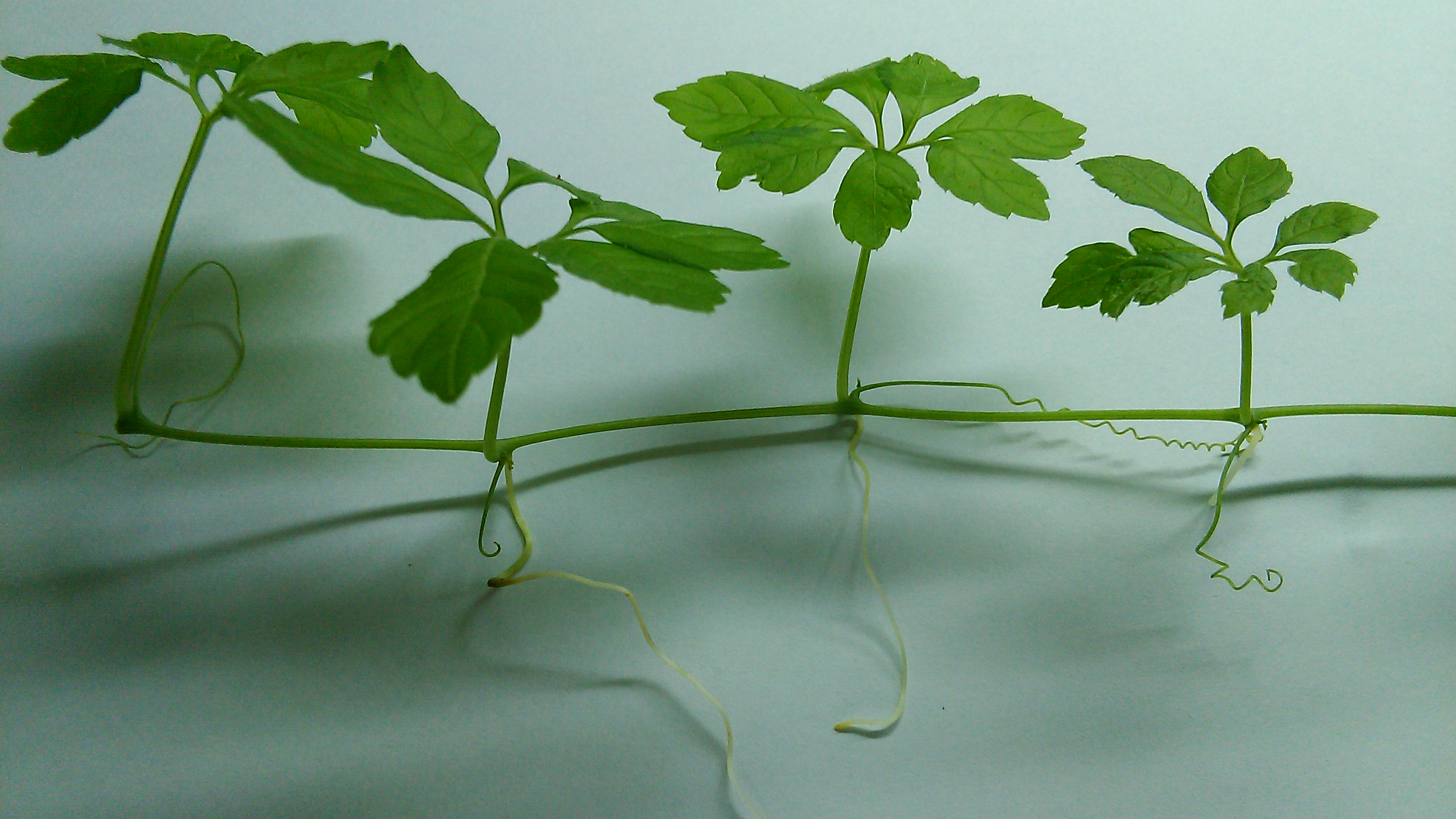

Supplement: Supplementary file 1 — Figure S1. G. pentaphyllum (Sample) 01. Description: This is the picture of intact G. pentaphyllum in our study. Figure S2. G. pentaphyllum (Sample) 02. Description: This is the picture of intact G. pentaphyllum in our study. Figure S3. Leaves (Sample). Description: This is the picture of leaves sample of G. pentaphyllum in our study. Figure S4. Stems (Sample). Description: This is the picture of stems sample of G. pentaphyllum in our study. Figure S5. Fibrous Roots (Sample). Description: This is the picture of fibrous roots sample of G. pentaphyllum in our study. Figure S6. The general result of annotation. Abbreviations: NR: Nonredundant protein sequences; GO: Gene Ontology; KEGG: Kyoto Encyclopedia of Genes and Genome; eggNOG: Evolutionary genealogy of genes: Nonsupervised Orthologous Groups. Figure S7. The result of GO Slim. Abbreviation: GO Slim: Cut-down versions of the GO ontologies. Figure S8. The result of eggNOG annotation. Figure S9. The result of KEGG annotation. Figure S10. The standard curve of absorbance. [file 7840914.f1.zip › Supplementary information/Figure S2. G. pentaphyllum (Sample) 02.jpg]

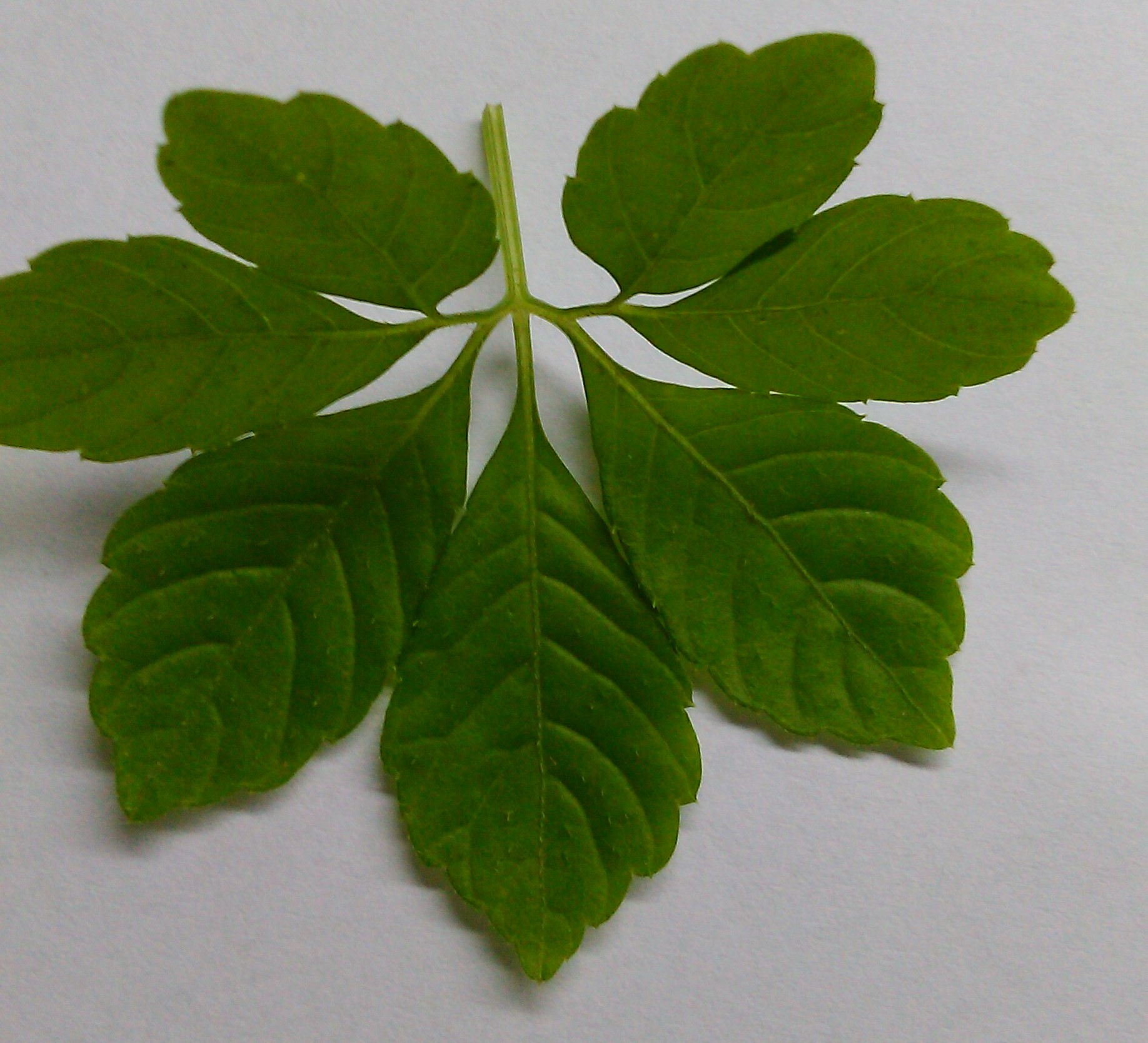

Supplement: Supplementary file 1 — Figure S1. G. pentaphyllum (Sample) 01. Description: This is the picture of intact G. pentaphyllum in our study. Figure S2. G. pentaphyllum (Sample) 02. Description: This is the picture of intact G. pentaphyllum in our study. Figure S3. Leaves (Sample). Description: This is the picture of leaves sample of G. pentaphyllum in our study. Figure S4. Stems (Sample). Description: This is the picture of stems sample of G. pentaphyllum in our study. Figure S5. Fibrous Roots (Sample). Description: This is the picture of fibrous roots sample of G. pentaphyllum in our study. Figure S6. The general result of annotation. Abbreviations: NR: Nonredundant protein sequences; GO: Gene Ontology; KEGG: Kyoto Encyclopedia of Genes and Genome; eggNOG: Evolutionary genealogy of genes: Nonsupervised Orthologous Groups. Figure S7. The result of GO Slim. Abbreviation: GO Slim: Cut-down versions of the GO ontologies. Figure S8. The result of eggNOG annotation. Figure S9. The result of KEGG annotation. Figure S10. The standard curve of absorbance. [file 7840914.f1.zip › Supplementary information/Figure S3. Leaves (Sample).jpg]

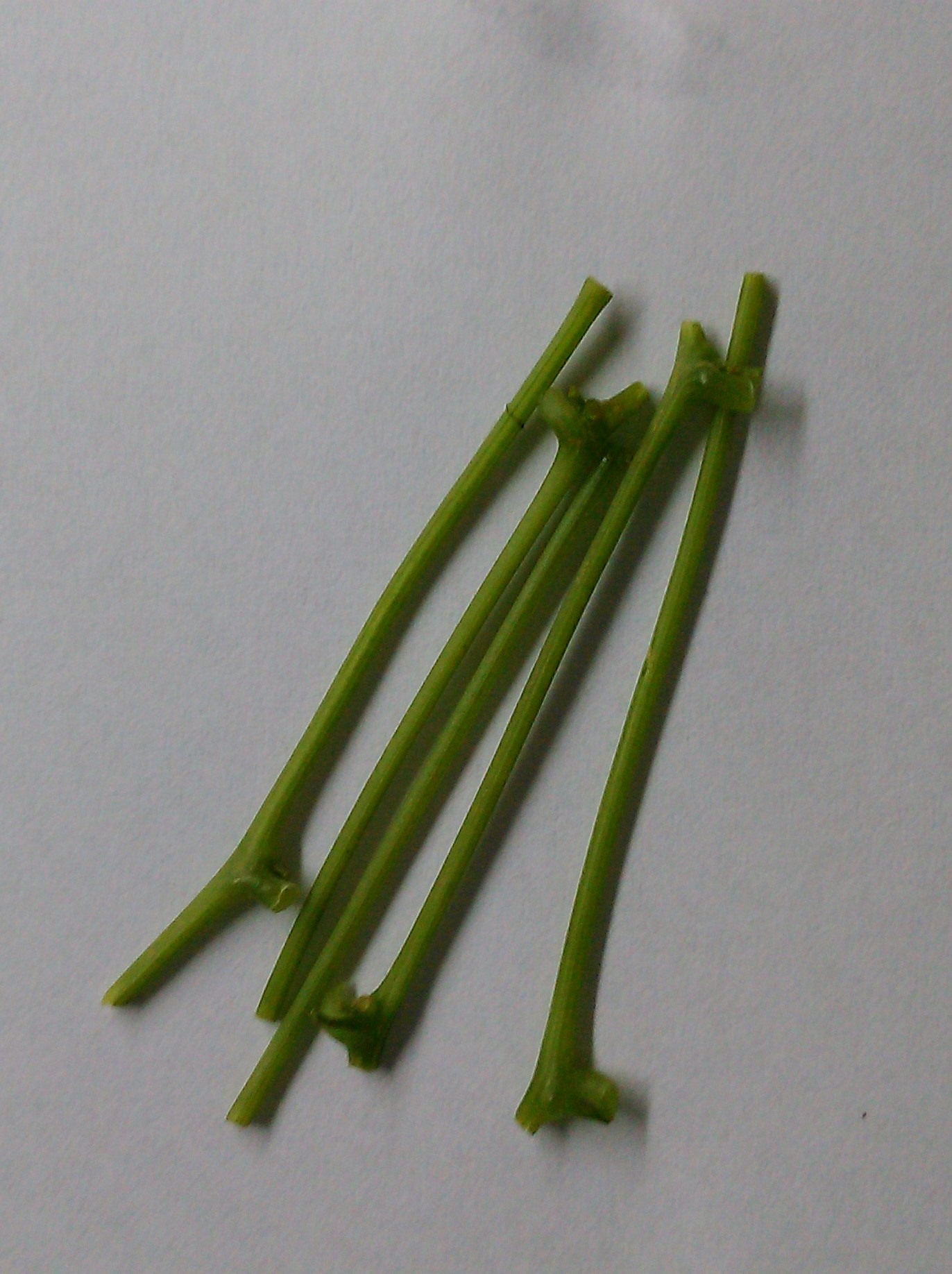

Supplement: Supplementary file 1 — Figure S1. G. pentaphyllum (Sample) 01. Description: This is the picture of intact G. pentaphyllum in our study. Figure S2. G. pentaphyllum (Sample) 02. Description: This is the picture of intact G. pentaphyllum in our study. Figure S3. Leaves (Sample). Description: This is the picture of leaves sample of G. pentaphyllum in our study. Figure S4. Stems (Sample). Description: This is the picture of stems sample of G. pentaphyllum in our study. Figure S5. Fibrous Roots (Sample). Description: This is the picture of fibrous roots sample of G. pentaphyllum in our study. Figure S6. The general result of annotation. Abbreviations: NR: Nonredundant protein sequences; GO: Gene Ontology; KEGG: Kyoto Encyclopedia of Genes and Genome; eggNOG: Evolutionary genealogy of genes: Nonsupervised Orthologous Groups. Figure S7. The result of GO Slim. Abbreviation: GO Slim: Cut-down versions of the GO ontologies. Figure S8. The result of eggNOG annotation. Figure S9. The result of KEGG annotation. Figure S10. The standard curve of absorbance. [file 7840914.f1.zip › Supplementary information/Figure S4. Stems (Sample).jpg]

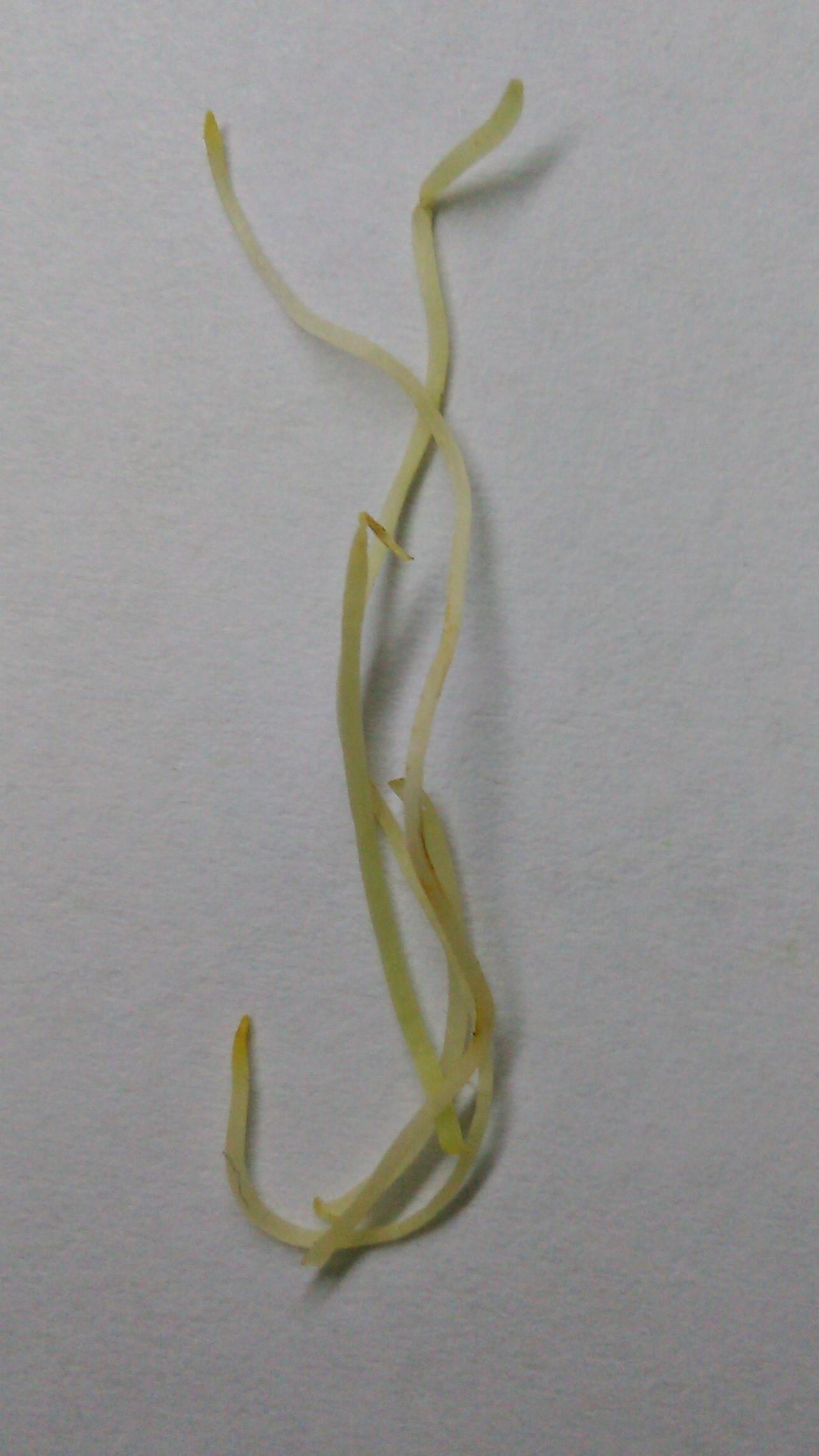

Supplement: Supplementary file 1 — Figure S1. G. pentaphyllum (Sample) 01. Description: This is the picture of intact G. pentaphyllum in our study. Figure S2. G. pentaphyllum (Sample) 02. Description: This is the picture of intact G. pentaphyllum in our study. Figure S3. Leaves (Sample). Description: This is the picture of leaves sample of G. pentaphyllum in our study. Figure S4. Stems (Sample). Description: This is the picture of stems sample of G. pentaphyllum in our study. Figure S5. Fibrous Roots (Sample). Description: This is the picture of fibrous roots sample of G. pentaphyllum in our study. Figure S6. The general result of annotation. Abbreviations: NR: Nonredundant protein sequences; GO: Gene Ontology; KEGG: Kyoto Encyclopedia of Genes and Genome; eggNOG: Evolutionary genealogy of genes: Nonsupervised Orthologous Groups. Figure S7. The result of GO Slim. Abbreviation: GO Slim: Cut-down versions of the GO ontologies. Figure S8. The result of eggNOG annotation. Figure S9. The result of KEGG annotation. Figure S10. The standard curve of absorbance. [file 7840914.f1.zip › Supplementary information/Figure S5. Fibrous Roots (Sample).jpg]
